# Central-variant posterior reversible encephalopathy syndrome in association with adrenal insufficiency: A case report

**DOI:** 10.1097/MD.0000000000041625

**Published:** 2025-02-21

**Authors:** Bahadar S. Srichawla, Vincent Kipkorir, Rakhee Lalla

**Affiliations:** a Department of Neurology, University of Massachusetts Chan Medical School, Worcester, MA; b Department of Medicine, University of Nairobi, Nairobi, Kenya.

**Keywords:** adrenal insufficiency, atypical PRES, brainstem, central variant, posterior reversible encephalopathy syndrome, PRES, reversible posterior leukoencephalopathy syndrome

## Abstract

**Rationale:**

Posterior reversible encephalopathy syndrome (PRES) is a neurotoxic condition often linked to hypertension, eclampsia, or renal failure. PRES typically presenting with seizures, headaches, visual disturbances, and altered mental status. A rarer form, the central variant of PRES, involves atypical radiologic findings such as edema in central brain structures. PRES has not been previously associated with adrenal insufficiency, making this case novel and significant.

**Patient concerns:**

A 59-year-old woman with a history of hypertension, chronic obstructive pulmonary disease, and previous COVID-19 infection presented to the emergency department with seizures and altered mental status. She exhibited a fluctuating systolic blood pressure (79–195 mm Hg) and had a Glasgow Coma Scale (GCS) score of 7.

**Diagnoses:**

Initial imaging and laboratory tests were inconclusive. Continuous electroencephalogram indicated focal cortical irritability, raising concerns about seizures. Brain magnetic resonance imaging revealed increased T_2_-weighted signals in the bilateral cerebellar hemispheres, consistent with central variant PRES. Endocrine evaluation showed primary adrenal insufficiency, confirmed by low AM cortisol levels and a positive cosyntropin stimulation test.

**Interventions:**

The patient was started on levetiracetam for seizure management and hydrocortisone for adrenal insufficiency. She was intubated for airway protection but later extubated as her condition stabilized.

**Outcomes:**

Follow-up magnetic resonance imaging showed progressive resolution of the cerebellar T_2_ hyperintensities. The patient was discharged on day 15 with no residual neurological deficits. At a 3-month follow-up, she remained seizure-free and continued oral hydrocortisone and levetiracetam.

**Lessons:**

This case highlights adrenal insufficiency as a possible novel precipitant of the central variant of PRES, emphasizing the need for prompt diagnosis and treatment to prevent serious neurological outcomes. The underlying pathophysiological mechanism of PRES from adrenal insufficiency is most likely labile blood pressure causing rapid alterations in cerebral perfusion pressure (CPP) precipitating PRES.

## 1. Introduction

Posterior reversible encephalopathy syndrome (PRES) is a neurotoxic state that typically presents with a range of clinical features, including seizures, headaches, visual disturbances, and altered mental status. This condition is characterized by vasogenic edema that predominantly affects the posterior regions of the brain, most often the parietal and occipital lobes. However, less commonly, PRES can affect the central structures of the brain (e.g., basal ganglia, thalamus, cerebellum, brainstem, etc) leading to what is known as the central variant of PRES.

The central variant of PRES, although rare, is particularly significant due to its atypical radiologic findings, which include edema in the basal ganglia, thalamus, brainstem, and periventricular white matter.^[1,2]^ These regions are less frequently affected in the classic presentation of PRES, making the central variant a diagnostic challenge. Understanding the underlying etiologies that contribute to this variant is crucial for appropriate management. We report the first case of central variant PRES that occurred secondary to adrenal insufficiency. The association between adrenal insufficiency and the development of PRES has not been previously reported in the scientific literature. By presenting this case, we hope to raise awareness among clinicians of the potential for adrenal insufficiency to precipitate PRES, particularly its central variant, and to emphasize the importance of timely diagnosis and intervention in such cases. This case report was completed under the established CARE guidelines for case reports/series.

## 2. Materials and methods

A 59-year-old woman with a medical history of COVID-19, hypertension, and chronic obstructive pulmonary disease presented to the emergency department with altered mental status and seizures. She had a decreased level of arousal and was intubated for airway protection in the emergency department. Her systolic blood pressure varied from 79 to 195 mm Hg within the first 6 hours of presentation. The neurological assessment showed an atraumatic woman with a Glasgow Coma Scale (GCS) of 7 (E_2_V_1_M_4_). Her Full Outline of Unresponsiveness (FOUR) score was 16 (E_4_M_4_B_4_R_4_). Her pupils were 3 mm equal and sluggishly reactive to light bilaterally. Deep tendon reflexes were 2+ all throughout without any pathological reflexes. Other brainstem reflexes including cough, gag, and corneal were present. The initial laboratory report including a comprehensive metabolic panel and complete blood count was unrevealing. A comprehensive toxicology screen was negative. Other laboratory tests including erythrocyte sedimentation rate, C-reactive protein were within normal limits. A noncontrast computerized tomography (CT) scan of the head and CT angiogram of the head and neck showed no acute intracranial abnormality, stenoses, or large vessel occlusion. The patient was admitted to the intensive care unit for further treatment.

**Figure 1.**
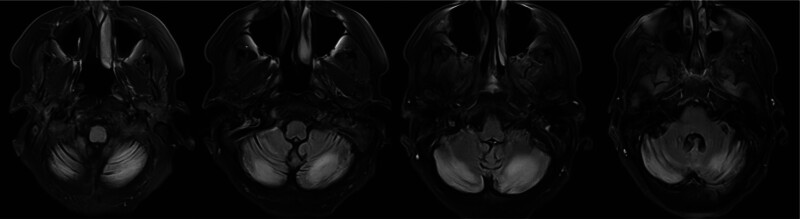
Magnetic resonance imaging of the brain with and without contrast showing an increased T_2_-weighted signal in the bilateral cerebellar hemispheres consistent with central variable PRES. PRES = posterior reversible encephalopathy syndrome.

A continuous electroencephalogram was completed for 72 hours showing frequent brief potentially ictal rhythmic discharges, bilateral generalized occasional sharp transients in the left posterior temporal lobe. There were also frequent blunted bifrontal generalized periodic discharges with triphasic morphology at 0.5 Hz. There was occasional lateralized rhythmic delta activity (LRDA) typically at 1.5 Hz within the left frontal and frontotemporal lobe. This pattern lies on the ictal-interictal continuum. Overall, electroencephalogram was concerning for focal cortical irritability with increased risk of seizures within the left frontotemporal and frontal lobes. She started on levetiracetam 1500 mg twice daily. Magnetic resonance imaging of the brain with and without contrast showed an increased T_2_-weighted signal in the bilateral cerebellar hemispheres consistent with vasogenic edema that spared the temporal and occipital lobes, raising concern for central-variant posterior reversible encephalopathy syndrome (PRES; Fig. 1). Other differential diagnoses included cerebellitis, primary central nervous system vasculitis, and cerebral venous sinus thrombosis. A lumbar puncture was performed, and cerebrospinal fluid (CSF) studies did not show abnormalities. The autoimmune and paraneoplastic antibodies panel of serum and CSF were negative. Further malignancy screening including chest, abdomen, and pelvic CT, ultrasound of the breast and ovaries were negative. Due to persistent fluctuations in blood pressure, endocrinology was consulted for further evaluation.

**Figure 2.**
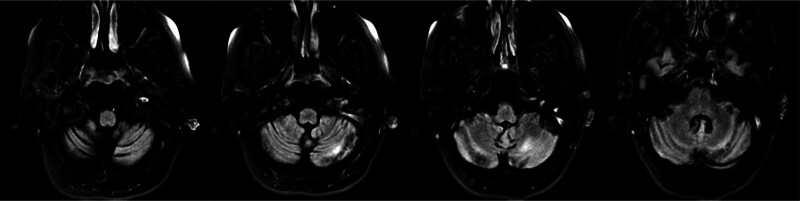
Magnetic resonance imaging of the brain with and without contrast showing improvement in the T_2_-weighted signal in the bilateral cerebellar hemispheres in 7 days.

**Figure 3.**
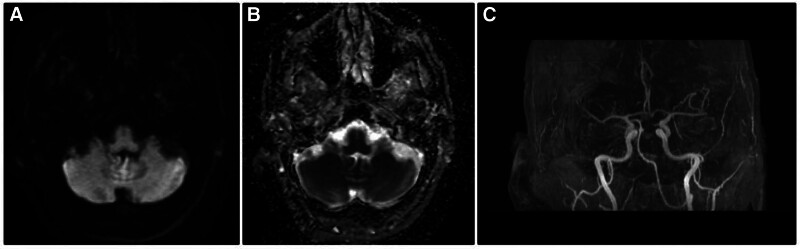
(A) Diffusion weighted imaging (DWI) showing no acute ischemia, (B) confirmed with apparent diffusion coefficient (ADC), (C) magnetic resonance angiography (MRA) showing no occlusion or narrowing. ADC = apparent diffusion coefficient, DWI = diffusion weighted imaging, MRA = magnetic resonance angiography.

She had a low AM cortisol of 0.8 µg/dL (6.7–22.6). A subsequent 60-minute cosyntropin stimulation test showed a positive result of <0.4 µg/dL, 7.0 µg/dL and 12.7 µg/dL at time points of 0, 30, and 60 minutes. Cortisol levels > 20 µg/dL at 30- and 60-minute are considered a normal response. She was diagnosed with primary adrenal insufficiency and was started on hydrocortisone 50 mg IV. Then a tapering oral dose of hydrocortisone was placed, and 15 mg PO was maintained in the morning and 5 mg at 1400 hours daily. The treatment of adrenal insufficiency aided in stabilizing the blood pressure. She was weaned from mechanical ventilation and extubated on day 5 of admission. A repeat brain magnetic resonance imaging 1 week later showed progressive resolution of cerebellar hyperintensities, and the patient was later extubated and had a favorable neurological outcome (Fig. 2). Magnetic resonance angiography of the head and neck with and without contrast was performed, showing no abnormalities (Fig. 3). Thus making, central nervous system vasculitis and cerebral venous sinus thrombosis less likely. She was discharged on day 15 of hospitalization to home with minor residual neurological deficits with a modified Rankin score (mRS) of 2. At 3 months of follow-up in the neurology clinic, she continues to be seizure free and remains on oral hydrocortisone and levetiracetam.

## 3. Discussion

In this case, we present the central variant of PRES secondary to adrenal insufficiency, a previously unreported and novel association that contributes to the growing diversity of conditions associated with this syndrome. PRES is a multifactorial disease with a wide range of underlying etiologies, including hypertension, eclampsia, renal failure, and autoimmune diseases. The pathophysiology of PRES is complex and not fully understood but is generally believed to involve endothelial dysfunction that leads to disruption of the blood-brain barrier and subsequent vasogenic edema.^[3,4]^

Adrenal insufficiency leads to a deficiency of glucocorticoids and mineralocorticoids, critical hormones for maintaining blood pressure, electrolyte balance, and vascular integrity.^[5]^ The resulting hypotension and electrolyte disturbances, such as hyponatremia and hyperkalemia, can profoundly affect cerebral perfusion and the stability of the blood-brain barrier.^[5]^ Adrenal insufficiency is often associated with hypotension due to decreased cortisol and aldosterone levels, which alter vascular tone and reduce intravascular volume.^[6]^ Rapid alterations in mean arterial pressure can lead to impaired cerebral autoregulation, predisposing to cerebral hypo- and hyperperfusion and subsequent vasogenic edema, particularly in the more vulnerable posterior regions of the brain. Severe hyponatremia, a common consequence of adrenal insufficiency, can lead to osmotic imbalances and intracellular swelling of neurons and glial cells. This osmotic shift exacerbates the development of vasogenic edema, particularly in regions with compromised vascular integrity.^[7]^ However, the patient reported here was not hyponatremic. Glucocorticoids have protective effects on the endothelium, including reducing permeability and modulating inflammatory responses. In adrenal insufficiency, the absence of these hormones may exacerbate endothelial dysfunction, further compromising the blood-brain barrier and promoting the formation of vasogenic edema.^[8]^ Adrenal insufficiency can disrupt the balance of the sympathetic nervous system, leading to an abnormal cerebral vascular response. This dysregulation may contribute to inappropriate vasoconstriction and vasodilation, leading to regional differences in blood flow and promoting the development of PRES.^[9]^ Additionally, adrenal insufficiency can cause decreased intravascular volume due to a deficiency in cortisol and aldosterone. This is the proposed pathophysiological mechanism causing PRES in our patient.

This case report is limited by the absence of direct mechanistic studies linking adrenal insufficiency to the central variant of PRES. Although the proposed mechanisms are based on established physiological principles and case reports, further research is needed to elucidate the precise pathways involved. Furthermore, this report is based on a single case, which may limit the generalizability of the findings. Although adrenal insufficiency was diagnosed in our patient it may have been an incidental finding and not the primary driver for blood pressure lability. Hyponatremia an objective finding in adrenal insufficiency was not seen in our case. Although no clear mechanism of hyponatremia related to PRES is established purported mechanisms may involve endothelial dysfunction or cerebral dysautoregulation. Future studies should aim to systematically investigate the incidence of PRES in patients with adrenal insufficiency, with particular attention to identifying risk factors that can predispose certain individuals to this complication. Furthermore, experimental models could be developed to explore the molecular and cellular mechanisms underlying the disruption of the blood-brain barrier in this context.

## 4. Conclusions

The central variant of posterior reversible encephalopathy syndrome (PRES) is a rare but clinically significant manifestation. We present the first case of PRES in association with adrenal insufficiency. This case highlights the importance of recognizing complications of adrenal insufficiency as a potential underlying mechanism associated with PRES, especially in patients with atypical radiologic findings and clinical symptoms. Pathophysiology most likely involves a combination of factors including rapid alterations in mean arterial pressure, electrolyte imbalances, and endothelial dysfunction, which together contribute to disrupting cerebral autoregulation and integrity of the blood-brain barrier. The prompt diagnosis and treatment of adrenal insufficiency are crucial to prevent the progression of PRES and to reverse its effects. Clinicians should maintain a high index of suspicion for PRES in patients with adrenal insufficiency who present with neurological symptoms, even in the absence of significant hypertension.

## Author contributions

**Conceptualization:** Bahadar S. Srichawla.

**Data curation:** Bahadar S. Srichawla, Vincent Kipkorir.

**Formal analysis:** Bahadar S. Srichawla.

**Funding acquisition:** Bahadar S. Srichawla.

**Investigation:** Bahadar S. Srichawla.

**Methodology:** Bahadar S. Srichawla.

**Project administration:** Bahadar S. Srichawla.

**Resources:** Bahadar S. Srichawla.

**Software:** Bahadar S. Srichawla.

**Supervision:** Bahadar S. Srichawla, Rakhee Lalla.

**Validation:** Bahadar S. Srichawla.

**Visualization:** Bahadar S. Srichawla.

**Writing – original draft:** Bahadar S. Srichawla.

**Writing – review & editing:** Bahadar S. Srichawla, Rakhee Lalla.

## References

[1] Chen DY Tseng YC Hsu HL Huang YL Chen CJ. Teaching neuroimages: central variant of posterior reversible encephalopathy syndrome. Neurology. 2014;82:e164. 24821938 10.1212/WNL.0000000000000407

[2] Hamid U Umair FA Nair D. Isolated infratentorial posterior reversible encephalopathy syndrome (PRES) in nephrotic syndrome: a case report. Cureus. 2024;16:e55056. 38550455 10.7759/cureus.55056 PMC10974883

[3] Fugate JE Claassen DO Cloft HJ Kallmes DF Kozak OS Rabinstein AA. Posterior reversible encephalopathy syndrome: associated clinical and radiologic findings. Mayo Clin Proc. 2010;85:427–32. 20435835 10.4065/mcp.2009.0590 PMC2861971

[4] Srichawla BS Quast J. Magnesium deficiency: an overlooked key to the puzzle of posterior reversible encephalopathy syndrome (PRES) and reversible cerebral vasoconstriction syndrome (RCVS)? J Neurol Sci. 2023;453:120796. 37708589 10.1016/j.jns.2023.120796

[5] Rafiq K Nakano D Ihara G. Effects of mineralocorticoid receptor blockade on glucocorticoid-induced renal injury in adrenalectomized rats. J Hypertens. 2011;29:290–8. 21243738 10.1097/hjh.0b013e32834103a9 PMC3034279

[6] Castle-Kirszbaum M Goldschlager T Shi MDY Fuller PJ. Glucocorticoids and water balance: implications for hyponatremia management and pituitary surgery. Neuroendocrinology. 2023;113:785–94. 37062279 10.1159/000530701 PMC10389798

[7] Largeau B Bergeron S Auger F. Experimental models of posterior reversible encephalopathy syndrome: a review from pathophysiology to therapeutic targets. Stroke. 2024;55:484–93. 38126184 10.1161/STROKEAHA.123.044533

[8] Welch MR. Management of complications in neuro-oncology patients. Continuum (Minneap Minn). 2023;29:1844–71. 38085901 10.1212/CON.0000000000001359

[9] Srichawla BS Presti K Kipkorir V Berrios Morales I. Chemotherapy-associated hemorrhagic posterior reversible encephalopathy syndrome (PRES) with considerations for circle of Willis variants on cerebral blood flow and autoregulation: a case report. Medicine (Baltim). 2024;103:e37250. 10.1097/MD.0000000000037250 PMC11309648 38394546

